# Structural and Spectroscopic Characterization of TiO_2_ Nanocrystalline Materials Synthesized by Different Methods

**DOI:** 10.3390/nano15070498

**Published:** 2025-03-26

**Authors:** Alise Podelinska, Elina Neilande, Viktorija Pankratova, Vera Serga, Hanna Bandarenka, Aliaksandr Burko, Sergei Piskunov, Vladimir A. Pankratov, Anatolijs Sarakovskis, Anatoli I. Popov, Dmitry V. Bocharov

**Affiliations:** 1 Institute of Solid State Physics, University of Latvia, LV-1063 Riga, Latvia; alise.podelinska@ut.ee (A.P.); elina.neilande@cfi.lu.lv (E.N.); viktorija.pankratova@cfi.lu.lv (V.P.); piskunov@cfi.lu.lv (S.P.); vladimirs.pankratovs@cfi.lu.lv (V.A.P.); anatolijs.sarakovskis@cfi.lu.lv (A.S.); anatolijs.popovs@cfi.lu.lv (A.I.P.); 2 Institute of Physics, University of Tartu, 50411 Tartu, Estonia; 3 Institute of Materials and Surface Engineering, Faculty of Materials Science and Applied Chemistry, Riga Technical University, LV-1048 Riga, Latvia; vera_serga@inbox.lv; 4 Applied Plasmonics Laboratory, Micro- and Nanoelectronics Department, Belarusian State University of Informatics and Radioelectronics, 220013 Minsk, Belarus; a.burko@bsuir.by

**Keywords:** titanium dioxide, extraction–pyrolytic, hydrothermal, sol–gel methods, rare earth elements doping, copper doping

## Abstract

Nanocrystalline materials based on titanium dioxide possess unique properties, including photocatalytic and antibacterial activities. Despite many approaches have already been utilized to fabricate and characterize pure and doped TiO_2_, a systematic description of its nanostructured samples depending on the synthesis method has not been presented yet. In this study, we shed new light on the process–structure relationships of nanocrystalline TiO_2_-based powders fabricated by extraction–pyrolytic, hydrothermal, and sol–gel techniques. The comprehensive analysis of the fabricated nanocrystalline TiO_2_-based powders with different anatase/rutile phase content is performed by scanning electron microscopy (SEM), energy-dispersive X-ray spectroscopy (EDX), Raman spectroscopy, and X-ray photoelectron spectroscopy (XPS). The hydrothermal and sol–gel methods are also used to grow TiO_2_ particles doped with Cu and Er-Yb. The correlation between synthesis parameters (pyrolysis and annealing temperature) and properties of the produced materials is studied. Particular attention is paid to Raman spectroscopy and the detailed comparison of our obtained data with existing experimental and theoretical studies.

## 1. Introduction

Titanium dioxide (TiO_2_) is a widely used prospective material with tremendous potential in numerous technological fields. This wide band gap semiconductor enables photocatalysis [1] and antibacterial activity [2], which are especially prominent in TiO_2_ nanostructures [3].

**Figure 1:**
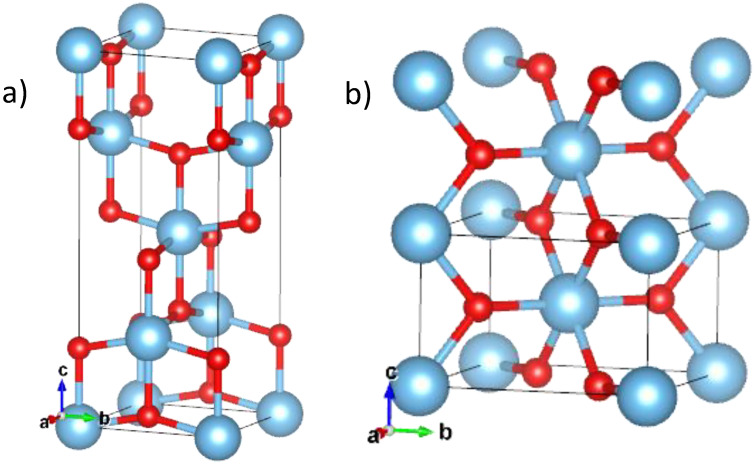
Anatase (**a**) and rutile (**b**) phases of titanium dioxide. Crystallographic cell borders are shown by black lines. Ti atoms are depicted in blue, while O atoms are shown in red, respectively. The structural properties of both compounds are collected in Table 1.

**Figure 2:**
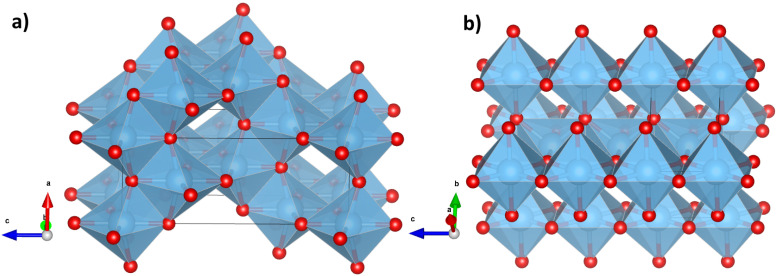
Polyhedral networks of (**a**) anatase and (**b**) rutile TiO_2_ phases. The blue spheres represent titanium atoms, while the red spheres represent oxygen atoms. The anatase network has longer Ti–O bonds and greater deviation from ideal octahedral angles, sharing four edges with neighboring octahedra to form a less dense, three-dimensional network without specific linear chains, resulting in a lower density. In contrast, the rutile octahedra feature shorter Ti–O bonds and angles closer to the ideal octahedral geometry, sharing two edges and two corners with neighboring octahedra to create linear chains along the c-axis, forming a more compact structure with a higher density.

The physicochemical properties and application areas of the TiO_2_ nanostructures can vary greatly and are closely related to their morphology. There are two main TiO_2_ polymorphic structural phases that attract the most attention for photocatalytic and antibacterial applications: metastable anatase and stable rutile [4,5,6]. Figure 1 shows a schematic representation of typical crystal cells for the anatase and rutile phases, while a description of their structural properties is given in Table 1. Both phases are characterized by tetragonal geometry but have different octahedron arrangements and interatomic distances [7]. The anatase form of titania has octahedral units of -TiO_6_- with longer Ti–O bonds and a greater deviation from the ideal octahedral shape. These octahedra share four edges with neighboring units, forming a less dense three-dimensional network free of distinct linear chains, resulting in a lower overall density. In contrast, the rutile form features octahedral units with shorter titanium–oxygen bonds and angles that are closer to the ideal octahedral geometry. The rutile octahedra share two edges and two corners with neighboring units, creating linear chains along the c-axis and a more compact structure of higher density (see Figure 1 and Figure 2 and Table 1). There are dissimilarities in the TiO_2_ lattice, yet the slightest differences in the elemental and chemical compositions can cause serious variability in optical and electronic properties, which are crucial for the functional parameters of TiO_2_-based materials [8]. TiO_2_ structure strongly depends on the technique used and the conditions of its fabrication.

Currently, the three most convenient fabrication approaches in terms of cost and technological procedures are preferably used for titanium dioxide synthesis: extraction–pyrolytic (EP), hydrothermal, and sol–gel methods. The EP method involves a combination of extracting components from aqueous solutions, mixing them, and pyrolyzing the pastes [9]. Hydrothermal synthesis is based on high pressure combined with heating, leading to the dissolution of reagents that are insoluble at room temperature and under atmospheric conditions [10]. The sol–gel method involves the dissolution of a molecular precursor in an organic solvent for sol formation followed by conversion to a gel induced by annealing [11].

Here, we report on a comparative analysis exploring the nanocrystalline TiO_2_ materials fabricated by the above techniques. The ultimate goal of this study was to investigate and elucidate the relationship between the synthesis conditions, morphology, and functional characteristics of the powders based on nanostructured TiO_2_. This was achieved through a systematic examination of how the different synthesis methods influence the structural and optical properties of the materials TiO_2_.

A comprehensive suite of characterization techniques was employed to assess the quality and properties of the synthesized TiO_2_ samples. These included scanning electron microscopy (SEM) to analyze surface morphology, energy-dispersive X-ray (EDX) spectroscopy for elemental analysis, X-ray photoelectron spectroscopy (XPS) to investigate chemical composition and oxidation states, and Raman spectroscopy for evaluating the vibrational properties and phase composition.

We also enriched the data pool collected for the pure TiO_2_ samples with those revealed for the Cu- and rare earth element (REE)-doped TiO_2_ synthesized by sol–gel and hydrothermal methods. The dopant types were selected because they can impart photocatalytic activity upon visible light excitation [12] and up-conversion [13] to TiO_2_ samples. Up-conversion luminescence describes the process in which two or more low-energy photons are absorbed and a single higher-energy photon is emitted. TiO_2_ doping with REEs and other up-conversion luminescence agents enables this phenomenon, thus activating TiO_2_ for photocatalysis at irradiation wavelengths in the near-UV range [14,15,16,17]. Thereby, the doped TiO_2_ opens new horizons for the development of radically new optical and antibacterial nanomaterials.

This research provides insights that can be used to optimize TiO_2_ synthesis for various technological applications, including photocatalysis and antibacterial treatments.

**Table 1:** Structural properties of anatase and rutile phase of titanium dioxide [18].

	Anatase	Rutile
Crystal structure	Tetragonal	Tetragonal
Space group	*I*$4_{1}$/amd	*P*$4_{2}$/mnm
	(Nº 141)	(Nº 136)
a = b (Å)	3.784	4.594
c (Å)	9.515	2.959
α = β = γ (°)	90	90
Density (${g/cm}^{3}$)	3.895	4.248

## 2. Experimental and Computational Details

### 2.1. Synthesis Methods

As mentioned above, we used three methods of TiO_2_ fabrication that are in high demand today. A detailed description of the methods and regimes used is given in the following subsections.

#### 2.1.1. Extraction–Pyrolytic Method

As a rule, the EP method is used to fabricate homogeneous nanocrystalline powders and films of oxide materials for various purposes. The EP process flow consists of two main steps: (i) preparation of a metal-containing precursor by liquid–liquid extraction using saturated fatty acids as extractors and (ii) subsequent thermal decomposition (pyrolysis) [9].

The precursor containing Ti was obtained by liquid extraction with the aqueous phase being a fresh 0.1 M solution of TiCl_3_ in hydrochloric acid (pH 0.5) and the organic phase consisting of n-pentanoic acid (valeric acid, C_4_H_9_COOH) without a diluent. The initial volume ratio of the aqueous and organic phases in the extraction system was 5:1. This ratio was defined in our earlier works, for example, in Ref. [19]. It allows increasing the titanium content in the organic phase for precursor preparation.

Liquid extraction was performed by adding a 1 M NaOH solution until the formation of a saturated solution of titanium valerate Ti(C_4_H_9_COOH) in valeric acid was achieved, i.e., when a finely dispersed precipitate was visually observed in the organic phase. After clear phase separation and removal of the aqueous phase, the organic phase was filtered through a double-thick paper filter. This resulted in the extraction of the Ti-containing precursor. The heat treatment (pyrolysis) of the aliquots of the precursor solution was carried out in the air through an increase in temperature from 21 °C to T_pyr_ ranging from 500 to 750 °C at a rate of 10 °/min. Once the T_pyr_ value was reached, the annealing lasted for 60 min. The process ended with rapid cooling of the TiO_2_ sample under ambient conditions.

#### 2.1.2. Hydrothermal Method

The hydrothermal method is a solution-based approach to produce nanostructured materials applied in electronics, biophotonics, biomedicine, and other fields with minimal product loss. The main principle of the hydrothermal method involves the dissolution of high-purity materials in water under high temperature and pressure, followed by the crystallization of the dissolved substance. The growing crystals and crystallites tend to reject contaminations from the synthesis environment, resulting in a high chemical purity of the material obtained [20].

In our study, we used the following conditions to fabricate TiO_2_ samples by the hydrothermal technique. A 0.12 M titanium isopropoxide (TTIP) solution in deionized water was prepared and stirred until a homogeneous state was reached. During stirring, hydrochloric acid was gradually added to the mixture to reach a 6 M HCl concentration in the solution. The concentration of HCl was selected based on the conditions of the TiO_2_ hydrothermal synthesis reported elsewhere [21]. This concentration can be considered rather high, which is necessary to keep the pH at a low level to control the TTIP hydrolysis kinetics and the geometrical parameters of the TiO_2_ nanoparticles. Doping of the sample TiO_2_ with Cu was performed by adding a copper salt (CuSO_4_) to the solution at a concentration of 2 mM. The REE-doping was carried out by introducing YbCl_3_/ErCl_3_ into the solution at a 2.8/0.28 mM ratio, respectively. The resulting solution was stirred and poured in a volume of 30 mL into a 50 mL Teflon cup installed in a steel autoclave. After that, the autoclave was closed and heated to 150 °C for 2 h and 45 min in a Nabertherm furnace to room temperature. After autoclaving, the solution with the pure TiO_2_ particles was dried in a ceramic crucible for 3 h at 70 °C followed by annealing at 500 °C for 30 min. The doped TiO_2_ samples were annealed at 400 °C for the same time.

#### 2.1.3. Sol–Gel Method

The sol–gel method belongs to the family of bottom-up approaches and represents a wet chemical technique for the synthesis of ceramic material (e.g., metal oxide or nitride) nanoparticles. The sol–gel method is implemented in a two-step procedure that includes (i) hydrolysis of the precursor in acidic or basic media followed by (ii) polycondensation of the hydrolyzed products [22].

To prepare TiO_2_ powders using the sol–gel method, titanium tetraisopropoxide (TTIP) was dissolved in 70% isopropanol to obtain a 0.4 mM TTIP solution, which was stirred until a homogeneous suspension formed. Then, deionized water was added to the suspension, and hydrolysis was carried out for 2 h. To obtain the doped TiO_2_ samples, copper sulfate (CuSO_4_) or REE salts, such as ytterbium chloride (YbCl_3_) and erbium chloride (ErCl_3_), were added to the solution at concentrations of 14 mM or 2.8/0.28 mM, respectively. After stirring, the resulting solution was left for a day to form a gel. An excess of the remaining liquid phase was removed from the gel. Subsequently, the gel was dried for 5 h at 80 °C. After that, the pure TiO_2_ sample was annealed at 400 °C for 30 min. The doped TiO_2_ samples were annealed at 500 °C for the same time.

The temperature variation applied in the final annealing stage for the pure and doped samples was intended to explain whether slight structural changes of TiO caused by heating can be detected.

The resulting TiO_2_ powders were collected from the crucible with a stainless steel laboratory spatula.

The rationale behind the selection of different annealing temperatures for pure TiO_2_ samples (400 °C) and doped TiO_2_ samples (500 °C) is connected to the activation of the dopant and the preservation of the crystalline structure of anatase. For example, in Ref. [23], the Ag-doped TiO_2_ with an anatase structure was synthesized even at a higher annealing temperature, at 600 °C, which led to a partial transformation to rutile if the TiO_2_ sample was undoped.

### 2.2. Characterization Details

#### 2.2.1. Raman Measurements

The Raman spectra of the TiO_2_-based samples were collected using the *TriVista 777* spectrometer (Princeton Instruments, Trenton, NJ, USA) with the 532 nm laser of 2.315 mW power. The exposure time for each sample was 1 s. All measurements were performed at a temperature of 295.15 K. The spectrometer was equipped with an upright Olympus microscope with an *Olympus UISe MPlanN* 100×/0.90 objective and a single continuous-wave frequency.

#### 2.2.2. SEM and EDX Spectrometry

The surface morphology and elemental composition of the TiO_2_-based powders were studied using SEM and EDX spectrometry with the *Thermo Scientific Helios 5 UX*. The SEM images were taken in the secondary electron mode with a through-the-lens detector.

#### 2.2.3. XPS Measurements

The chemical composition of the experimental samples was characterized by XPS. XPS measurements were performed using the ThermoFisher *ESCALAB Xi+* instrument with a monochromatic Al Kα X-ray source (hν=1486.6 eV). The calibration of the binding energy scale was confirmed by examining the reference samples of Au, Ag, and Cu cleaned with sputter, which placed Au 4f_7/2_, Ag 3d_5/2_, and Cu 2p_3/2_ peaks at 84.0 eV, 368.2 eV, and 932.6 eV, respectively. Spectra were recorded using an X-ray beam of 650 × 100 µm spot size with a pass energy of 20 eV and a step size of 0.1 eV. A pass energy of 150 eV and a step size of 1 eV were used to record the survey spectra. In the reported experiments, a charge neutralizer was used. All spectra were referenced to an adventitious carbon line to appear at 284.8 eV. Prior to the XPS measurements, the TiO_2_ powders were pressed into tablets.

### 2.3. Computational Details

The density functional theory (DFT) calculations of Raman spectra were performed using the Crystal23 code [24]. The PBE0 [25,26] hybrid version of the PBE functional [27], incorporating 25% Hartree–Fock exchange, was chosen for its time efficiency and recommendation by the Crystal23 code authors [26]. For the calculation of both TiO_2_ polymorphs (anatase and rutile), the 2 × 2 × 3 supercell was used. The optimized basis set for TiO_2_ was chosen form Piskunov et al.’s previous work [28]. Using Pack–Monkhorst/Gilat shrinking factors [24] and an 8 × 8 × 8 shrinking factor, the reciprocal space was sampled, yielding 105 points for rutile and 150 points for anatase in the irreducible region of the Brillouin zone. For geometry optimization and phonon frequency calculations, the self-consistent field convergence on the difference in total electronic energy was set to $10^{-7}$ hartree and $10^{-9}$ hartree, respectively.

## 3. Results and Discussion

### 3.1. Raman Study of the TiO_2_–Based Structures

Raman spectra analysis is a powerful characterization technique that provides in-depth information on the crystalline lattice structure and molecular composition of the TiO_2_-based samples. Raman spectroscopy has often been reported as a method used to study titanium dioxide samples containing impurities, including lanthanides [29,30,31].

Here, we turn to both theoretical and experimental approaches for the complementary Raman study of titanium dioxide. Table 2 compiles data on the band gap, bound distances, and lattice parameters of the theoretical models.

The characteristic peaks for anatase and rutile are presented in Table 3. The Raman spectrum of anatase TiO_2_ exhibits several active modes, including the prominent E_g_ mode with peaks around 144, 197, and 640 cm^−1^ [32,33] resulting from the symmetric stretching vibration of O–Ti–O in TiO_2_ [34]. The E_g_ mode is sensitive to particle size, showing a blueshift and a broadening when nanoparticles are studied [35,36]. This size-dependent behavior is attributed to the three-dimensional confinement of phonons in finite-sized nanocrystals [37]. Other notable modes are the B_1g_ mode with peaks around 519 and 399 cm^−1^, and the A_1g_ mode with a peak around 513 cm^−1^ [32,33]. The mentioned A_1g_ and B_1g_ peaks around 515 cm^−1^ commonly form the doublet where B_1g_ relates to the symmetric bending vibration of O–Ti–O, and A_1g_ relates to its antisymmetric bending vibration [38]. Furthermore, defects and stoichiometry also influence the Raman spectra. Iida and coauthors [36] reported that Raman spectra are more sensitive to defects than XRD spectra.

The Raman spectrum of rutile TiO_2_ exhibits peaks around 144, 447, 612, and 826 cm^−1^, corresponding with the vibrational modes B_1g_, E_g_, A_1g_, and B_2g_, respectively [39,40]. In addition, the B_1g_ mode exhibits intriguing characteristics, including its relatively weak temperature dependence and broadening that can be attributed to the mode’s quartic anharmonicity and anomalous phonon–phonon interactions [36]. The rutile spectrum also exhibits a distinct feature of pronounced second-order Raman scattering occurring around 240 cm^−1^ [41].

DFT calculations were performed to acquire Raman spectra of the bulk TiO_2_ in the anatase and rutile phases as references for the further experimental Raman interpretation of the TiO_2_ samples formed by different techniques [40].

**Table 2:** Band gap ($E^{\mathrm{gap}}$), lattice parameters (*a* and *c*), and bond distances (*d*) for the fully relaxed TiO_2_ structure calculated using the hybrid functional PBE0 [25] and the computational code Crystal23 [24]. The parameter $\alpha^{\mathrm{HF}}$ represents the fraction of Hartree–Fock exchange. The calculated values are compared with the experimental data.

	Polymorph	$\alpha^{\mathrm{HF}}$	*a*, Å	*c*, Å	$d^{\mathrm{TiO}}$, Å	$d^{\mathrm{TiTi}}$, Å	$d^{\mathrm{OO}}$, Å	$E^{\mathrm{gap}}$, eV
Crystal23	anatase	0.25	3.779	9.553	1.9336	3.0452	2.4536	4.13
					1.9768	3.7786	2.7958	
	rutile	0.25	4.586	2.964	1.9455	2.9636	2.5213	3.93
					1.9822	3.5654	2.7775	
					3.4793		2.9636	
Experiment	anatase		3.785 [42]	9.511 [42]				3.21 [43]
	rutile		4.592 [44]	2.958 [44]				3.00 [43]

#### 3.1.1. DFT Calculations of Bulk TiO_2_ Raman Spectra

**Figure 3:**
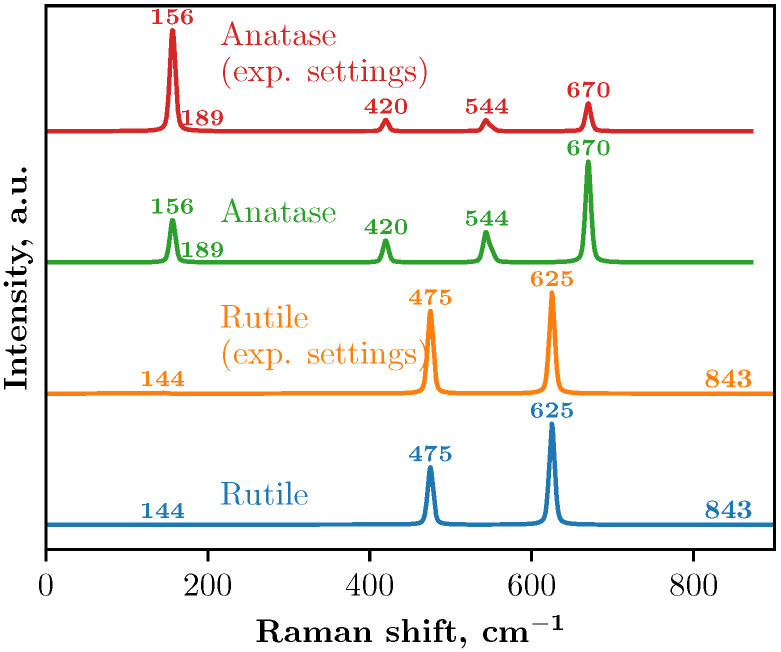
Raman spectra of 2 × 2 × 3 TiO_2_ bulk supercell calculated with the Crystal23 [24] code.

Raman spectra using a 2 × 2 × 3 supercell obtained from the DFT calculations are shown in Figure 3. Two approaches were used to simulate the Raman spectra: with and without the experimental setup.

In Crystal23, the keyword RAMANEXP involves the modification of the Raman intensity formula to account for experimental conditions. This model ensures that the intensities of the vibration modes match the experimental observations by incorporating the Bose distribution, account for the wavelength-dependent scattering efficiency, and average over orientations for comparison with powder sample experiments [26]. In our calculations with the experimental setup, the temperature was set to 298.15 K and the wavelength to 525 nm.

In the Raman spectra of anatase, both calculations with and without the experimental setup yield identical peak positions, although the intensities differ. Five main peaks were identified in both cases (see Table 3 and Figure 3) and theoretical calculations incorporating the experimental setup produce peak intensities that align more closely with the experimental data obtained in this study and previous studies [32,33,35,39,45]. The Raman spectra also correspond well with the study of Taudul et al. [34]. This study employed Crystal17 software with localized all-electron basis sets to calculate the vibrational properties and Raman spectra of anatase TiO_2_. The calculations were performed using different exchange-correlation functionals, including PBE, PBEsol, PBE-D3, B3LYP, and PBE0, in combination with the DZVP and TZVP basis sets. Both basis sets include d-polarization functions for Ti and O atoms and f-polarization for Ti atoms. The PBE functional tends to underestimate frequencies, while PBE0, PBEsol, and B3LYP provide values closer to the experimental results. In another study [46], the computations were performed with the ABINIT code, using pseudopotentials and a plane-wave basis set. The local density approximation (LDA) was applied for the exchange-correlation energy, and Teter-type extended norm-conserving pseudopotentials were used for titanium and oxygen.

Additionally, the vibrational properties for both TiO_2_ polymorphs were investigated using first-principles DFT within the VASP by Frank et al. [41]. The LDA and projector-augmented wave method were employed to model the electronic structure. The isotopic substitution of oxygen atoms was considered by adjusting the mass matrices, enabling the prediction of isotope-dependent frequency shifts. The calculated frequencies were validated by comparing them with the experimental Raman spectra acquired at temperatures as low as 5 K.

A similar pattern is observed in the Raman spectra of the rutile crystal: both calculations identify the same peak positions but with differing intensities. In this case, four peaks were identified (see Table 3). These results correlate well with Ref. [40], where computational modeling of the Raman spectra for the rutile polymorph was carried out using the GULP software package and the Buckingham interatomic potential, and the phonon frequencies and line widths were determined through the Fourier transform of the velocity autocorrelation function, as well as with Ref. [47] where Raman spectroscopy characterization of rutile nanocrystals embedded in cluster-assembled TiO_2_ films was performed. Separately, we note that some peaks on the graph have low intensity, and their presence was verified through the analysis of the Raman active modes listed in the output of the Crystal23 calculations.

**Table 3:** Identification of calculated anatase and rutile bulk Raman spectra peaks and comparison with previous experimental measurements of anatase and rutile bulk.

Phase	Calculated, cm^−1^	Calculated in Another Study, cm^−1^	Experimental Values	Assignment
Anatase	156.5	122.97–146.51 [34], 145.6 [46]	144 [32], 143 [33], 144 [35], 147 [39], 144 for $O^{16}$ [41], 144 [45]	E_g_ [32,41,45]
	189.5	169.88–194.66 [34], 171.1 [46]	197 [32], 198 [33], 200 [35], 198 [39], 196 for $O^{16}$ [41], 197 [45]	E_g_ [32,41,45]
	419.6	368.84–413.70 [34], 398.4 [46]	399 [32], 395 [33], 397 [35], 398 [39], 394 for $O^{16}$ [41], 400 [45]	B_1g_ [32,41,45]
	550.6	497.84–535.76 [34], 518.4 [46]	519 at 73 K [32], 512 [33], 516 [35], 515 [39], 516 for $O^{16}$ [41], 515 [45]	B_1g_ [32,41,45]
	543.3	486.79–524.54 [34], 535.9 [46]	513 at 73 K [32], 518 [33], 516 [35], 515 [39], 516 for $O^{16}$ [41], 519 [45]	A_1g_ [32,41,45]
	669.9	606.67–646.00 [34], 662.1 [46]	639 [32], 639 [33], 639 [35], 640 [39], 638 for $O^{16}$ [41], 640 [45]	E_g_ [32,41,45]
Rutile	143.7	169 [40], 141.2 for $O^{16}$ [41]	144 [39], 143 [40], 145 for $O^{16}$ [41], 143 [48], 142 [49]	B_1g_ [39,40,41]
	474.9	400 [40], 466.3 for $O^{16}$ [41]	448 [39], 447 [40], 448 for $O^{16}$ [41], 447 [48], 439 [49]	E_g_ [40,41,50]
	625.0	558 [40], 614.6 for $O^{16}$ [41]	612 [39,40,48], 613 for $O^{16}$ [41], 608 [49]	A_1g_ [39,40,41,50]
	843.2	803 [40], 819.1 for $O^{16}$ [41]	827 [39], 826 [40], 826 for $O^{16}$ [41], 826 [48], 810 [49]	B_2g_ [39,40,41]

#### 3.1.2. Raman Spectroscopy of the Pure TiO_2_ Samples

**Figure 4:**
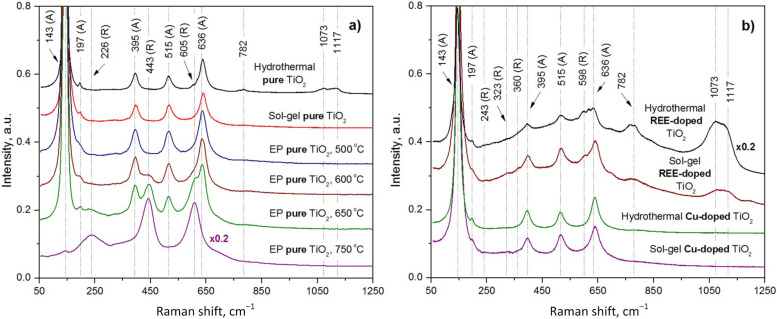
Measured Raman spectra of the pure (**a**), and Cu- and REE-doped (**b**) TiO_2_ samples formed by the extraction–pyrolytic, hydrothermal, and sol–gel methods.

The experimentally measured Raman spectra of the pure TiO_2_ samples synthesized by the EP, hydrothermal, and sol–gel methods are shown in Figure 4a.

We investigated four samples synthesized by the EP method at different annealing temperatures to reveal a heating effect on the TiO_2_ phase and molecular composition. The sample annealed at the lowest temperature (500 °C) comprises only the anatase phase with the corresponding peaks at 143, 197, 395, 517, and 636 cm^−1^ (see Table 3). The increase in temperature led to a gradual conversion to the rutile phase as seen in Table 4. In more detail, the TiO_2_ sample annealed at 600 °C is characterized by the Raman spectrum with anatase peaks (143, 192, 395, 515, and 634 cm^−1^) and the peak of rutile E_g_ at 440 cm^−1^. This indicates a small amount of the rutile crystallites in the sample. (It should be specifically noted that the B_1g_ rutile peak near 145 cm^−1^ cannot be used for the verification of phase transformations, as it is obscured by the much more intense anatase E_g_ peak located in the same region). The Raman spectrum of TiO_2_ heated to 650 °C also contains the characteristic bands of anatase, but peaks are observed at 226 (second-order Raman scattering [41]), 443 (E_g_), and 605 (A_1g_) cm^−1^, which means a tendency to formation of the rutile phase. At the highest annealing temperature (750 °C), the anatase phase completely transformed into the rutile one.

The temperature dependence of the structure of the sol–gel and pure hydrothermal TiO_2_ samples has already been reported elsewhere [51,52], unlike the EP TiO_2_ samples, which have been studied to a lesser extent. Therefore, we avoided repeating the determination of the temperature effect on the Raman spectra of the pure TiO_2_ samples formed by the sol–gel and hydrothermal methods. It should be noted that the transition from anatase to rutile, associated with an increase in annealing temperature, is the same for TiO_2_ formed by each of the methods, as shown by a comparison of our data for the EP TiO_2_ sample and the previously reported results for hydrothermal and sol–gel TiO_2_ samples.

The pure sol–gel TiO_2_ annealed at 400 °C exclusively contains the anatase phase, as evidenced by peaks at 144, 196, 395, 518, and 639 cm^−1^.

A slight increase in the annealing temperature to 500 °C of a pure hydrothermal TiO_2_ resulted, as expected, in a small rutile content expressed by an additional Raman band at 605 cm^−1^.

Weak peaks at 782, 1073, and 1117 cm^−1^ were detected exclusively in the hydrothermal TiO_2_ sample, but not in those synthesized by the sol–gel or extraction–pyrolytic methods, underscoring their uniqueness to the hydrothermal synthesis. According to the literature, the peaks around 782, 1073, and 1117 cm^−1^ correspond to vibrations of the C–H, C–O, and C–C groups [53,54,55,56], which can be caused by the adsorption of organic molecules on the TiO_2_ surface from air, as well as signifying that the samples are not completely washed of products of TTIP decomposition. These peaks can be indirect evidence of differences in the surface structure of the samples. In particular, the hydrothermal TiO_2_ sample is likely to have a higher particle package density and larger specific surface area, capable of holding or capturing undesirable contaminations from the reaction solutions and environment, while the sol–gel and EP TiO_2_ samples supposedly have similar surface morphology.

#### 3.1.3. Raman Spectra Analysis of REE-Doped TiO_2_ Samples

In this study, we modified the TiO_2_ samples with dopants of REEs (Yb and Er) and Cu (as observed in the next subsection). The Raman spectra of pure sol–gel, hydrothermal, and EP samples annealed at 400–500 °C were found to have nearly the same Raman band positions in the 300–700 cm^−1^ range. However, the hydrothermal sample is characterized by additional Raman bands at larger wave numbers, which means that its structure differs from those of the sol–gel and EP samples. Therefore, the effect of doping was studied only in sol–gel and hydrothermal TiO_2_ samples.

The Raman spectra of the REE-doped TiO_2_ samples reveal the presence of both the anatase and rutile phases, regardless of whether the samples were synthesized using the hydrothermal or sol–gel method (see Table 5).

For the sol–gel samples annealed at 500 °C, two different REE Yb:Er ratios were investigated—one sample had a Yb:Er volume ratio of 17.3:1, while the other had a ratio of 17.4:1. Initially, we expected to observe a redshift in the Raman spectrum peaks with an increase in the REE-dopant concentration, but the Raman spectra of the two samples were nearly identical.

Thereby, we presented only the Raman spectrum of the REE-doped TiO_2_ sample with a 17.3:1 volume ratio of Yb and Er in Figure 4. The peaks of the anatase phase are observed at 146, 196, 398, 517, and 640 cm^−1^, while the peaks of the rutile phase are located at 243, 323, 360, and 598 cm^−1^.

A Ti–OH vibration peak is identified at 702 cm^−1^. The peaks attributed to the vibrations of the C–H, C–O, and C–C groups [53,54,55] are observed at 770, 1093, and 1124 cm^−1^. The presence of rutile peaks indicates that an increased annealing temperature (500 °C vs. 400 °C for the pure sample) initiated the transformation of the anatase phase into rutile.

For the hydrothermal REE-doped sample (Yb:Er = 17.5:1), Raman analysis revealed anatase peaks at 144, 196, 395, 518, and 633 cm^−1^, and rutile peaks at 237 and 597 cm^−1^. A peak at 701 cm^−1^ is attributed to Ti–OH vibrations. The peaks at 769 cm^−1^, 1076, and 1115 cm^−1^ corresponding to C–H, C–O, and C–C vibrations show an increased intensity compared to the sol–gel REE-doped sample. This must be caused by a larger amount of organic contaminants remaining in the hydrothermal sample due to a lower annealing temperature.

In general, Raman spectra reveal a higher overall intensity for hydrothermal REE-doped TiO_2_ compared to sol–gel samples, with a lower rutile phase concentration due to a higher amount of REE-dopants. Both synthesis methods show higher rutile phase concentrations in REE-doped samples than in the pure TiO_2_ powders. However, higher REE concentrations reduce the rutile phase content, which is consistent with that of sol–gel REE-doped samples.

Additionally, the hydrothermal powder shows significantly stronger peaks at 769, 1076, and 1115 cm^−1^, indicating a higher concentration of organic contaminations compared to the sol–gel sample.

#### 3.1.4. Raman Spectra Analysis of Cu-Doped TiO_2_ Samples

The Cu-doped TiO_2_ samples synthesized by hydrothermal and sol–gel methods were also characterized by Raman spectroscopy (see Table 6).

The Raman spectrum of the sol–gel Cu-doped TiO_2_ sample showed only the peaks of the anatase phase at 147, 195, 396, 515, and 640 cm^−1^. Similarly, the Cu-doped TiO_2_ sample obtained by the hydrothermal method exhibited only the anatase phase with corresponding peaks at 145, 197, 395, 515, and 638 cm^−1^.

The intensity of the Raman spectrum is higher in the hydrothermal Cu-doped TiO_2_ sample compared to the sol–gel sample, which matches the results obtained for both the pure and REE-doped TiO_2_ samples. We suppose that it can be caused by the higher packing density of the hydrothermal TiO_2_ in contrast to the sol–gel method.

In both the sol–gel and hydrothermal Cu-doped TiO_2_ samples, only the anatase phase is visible. These results for the Cu-doped TiO_2_ samples contrast with the findings for the pure and REE-doped TiO_2_ samples, where rutile phase peaks were revealed. Rutile phase peaks were observed only in the hydrothermal method for pure TiO_2_ samples and in both the sol–gel and hydrothermal methods for REE-doped TiO_2_ samples. In the case of the sol–gel REE-doped TiO_2_ sample, the rutile phase appears due to the higher annealing temperature of the sample compared to the pure sol–gel TiO_2_ sample. The absence of rutile phase peaks in the Cu-doped TiO_2_ samples can be attributed to a combination of doping effects rather than solely annealing conditions. One possible explanation for why Cu specifically inhibits rutile phase formation is that Cu ions stabilize anatase by affecting the crystallization process, likely due to their ionic radius and interaction with the TiO_2_ lattice. Cu doping may induce lattice strain or grain boundary effects that suppress the anatase-to-rutile transition, whereas REE doping promotes it, possibly due to differences in ionic size and charge compensation mechanisms.

Furthermore, in the case of the Cu-doped TiO_2_ samples, the C–H, C–O, and C–C group peaks are absent for both sol–gel and hydrothermal samples. For pure TiO_2_ powders, these peaks were observed only in the hydrothermal sample, while REE-doping resulted in the formation of the powders with organic residues in both sol–gel and hydrothermal samples. The intensity of the corresponding Raman peaks was more intensive for the hydrothermal powder than for the sol–gel one.

### 3.2. Characterization of the TiO_2_ Morphology by SEM

**Figure 5:**
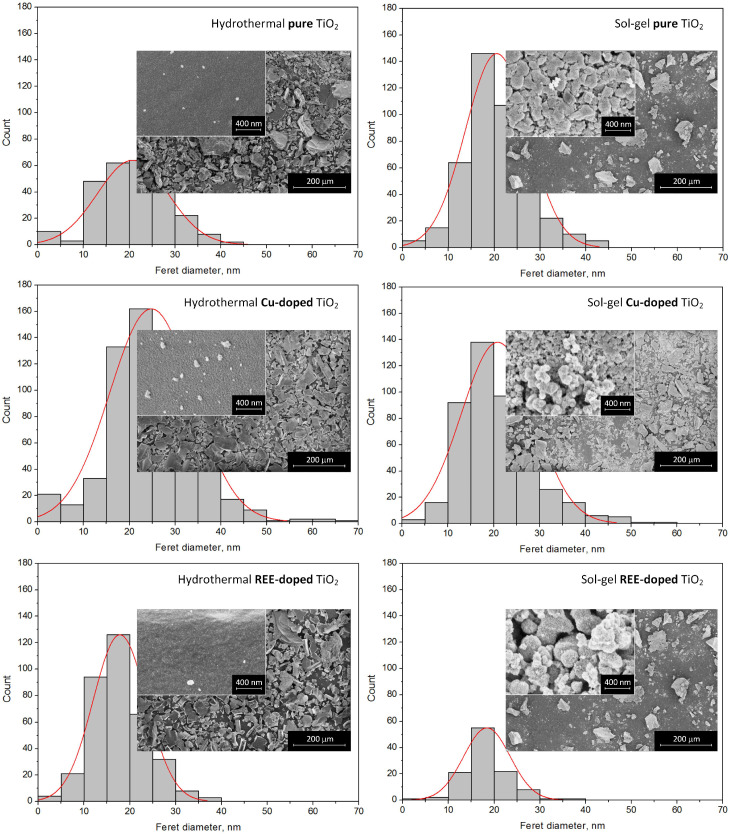
SEM images and corresponding size distribution histograms of the pure and Cu- and REE-doped TiO_2_ particles formed by hydrothermal (**left** column) and sol–gel (**right** column) methods. Magnification: 250× and 100,000× (insets).

All samples after synthesis and collection from laboratory glassware were powders with different dispersions. At the same time, hydrothermal powders were visually finer than sol–gel ones. Due to the fine dispersion, the hydrothermal powders were also denser. This observation is confirmed by the results of SEM analysis. The differences in morphology of the TiO_2_ particles synthesized by sol–gel and hydrothermal methods were revealed, as illustrated in Figure 5 where the SEM images and histograms of the particle size distribution are shown. It is seen that both hydrothermal and sol–gel powders consist of large agglomerates of 10 to 150 µm (see SEM images with a scale of 200 µm). At the same time, many agglomerates in hydrothermal samples have the form of plates with sharp edges and tips. Sol-gel agglomerates, in contrast, have a more rounded shape. The packing density of hydrothermal powders, as expected, is higher than that of sol–gel powders. Higher resolution SEM analysis of the powders showed that the agglomerates consist of nanoparticles ranging in size from a few nanometers to 70 nm (see SEM images with a scale bar of 400 nm and histograms). It is important to note that the hydrothermal agglomerates have a more uniform surface area on their faces, and the nanoparticles are densely packed in them. In the subsection on Raman analysis, we assumed that these structural features may result in favoring the adsorption of foreign molecules from the environment. We saw that the presence of Raman bands in the region of 700–1200 ${cm}^{-1}$ was not characteristic of all hydrothermal samples. Copper-doped titanium oxide synthesized hydrothermally does not contain peaks in this range, nor does the sol–gel one. On the other hand, both hydrothermal and sol–gel REE-doped powders have strongly pronounced Raman bands corresponding to the C–H, C–O, and C–C groups. The same samples are characterized by larger nanoparticle sizes up to 60–70 nm in contrast to the maximum 40–45 nm of the pure and copper-doped samples. At the same time, the pure hydrothermal sample with low-intensity Raman bands in the 700–1200 ${cm}^{-1}$ range also has a slightly larger nanoparticle size compared to the pure sol–gel and copper-doped samples. This follows from its size histogram, which has a lower intensity of bars for sizes less than 20 nm. It can be concluded that samples with larger nanoparticles tend to collect molecules from the environment on their surface, which are harder to wash away from chemical reaction products. On the contrary, it is generally accepted that smaller structures should better adsorb molecules due to their large specific surface area. However, in this case, the size of pores between nanoparticles, which is larger and allows the capture of high molecular compounds, is obviously of dominant importance. Also, apparently, the by-products of the reactions are inside the larger nanoparticles, being washed out from the surface only, while smaller nanoparticles with a larger specific area are cleaned much better during washing.

### 3.3. EDX Elemental Analysis of TiO_2_ Samples

**Figure 6:**
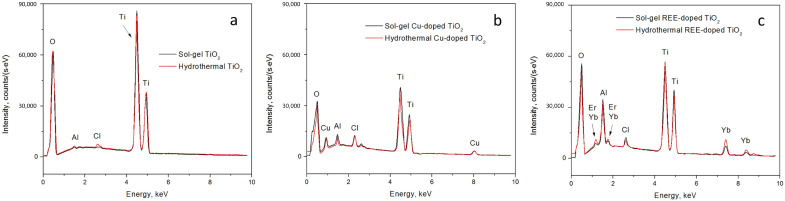
EDX-spectra of the (**a**) pure and (**b**) Cu- and (**c**) REE-doped TiO_2_ particles formed by the hydrothermal (red lines) and sol–gel (black lines) methods.

Then, the EDX spectra of both the pure and doped sol–gel and hydrothermal TiO_2_ samples were collected (Figure 6). The intensity of peaks in EDX spectra reflects both the relative quantity of specific atoms in the sample and the distribution of elements within the material, such as the depth at which different dopants are located. This is particularly interesting for comparisons of the TiO_2_ samples synthesized by sol–gel and hydrothermal methods.

In the case of pure TiO_2_ synthesized through the sol–gel method, we observed a characteristic oxygen (O) peak at approximately 0.5 keV, two titanium (Ti) peaks at around 4.5 keV and 5 keV, and peaks at approximately 1.5 keV and 2.7–2.8 keV, which are not typical for TiO_2_. Identifying the origin of these unknown peaks has proven challenging, as no prior publications provide a precise identification of these peaks in EDX spectra of pure TiO_2_. However, certain studies have attributed these peaks to aluminum (Al) [57,58,59] and chlorine (Cl) [60,61,62]. As a rule, the peaks of aluminum in the EDX spectra are neglected, since they are caused by the signal from the pin stubs for the sample mounting in the SEM chamber. The presence of chlorine in the samples may be due to the use of hydrochloric acid in one of the processes of titanium oxide synthesis. Chlorine is probably not completely washed out of the hydrothermal samples. In the sol–gel samples, chlorine is most likely present due to its adsorption from the air in the fume hood.

When examining the TiO_2_ sample synthesized by the sol–gel method with REE dopants, we observed that the peaks at approximately 1.8 keV, 7 keV, and 7.5 keV corresponded to Yb and Er. The peak around 1.8 keV is commonly associated with either erbium (Er^3+^) or ytterbium (Yb^3+^). Consequently, Er^3+^ is expected at approximately 7 keV, and Yb^3+^ should be detected around 7.5 keV [63,64,65].

In the context of the TiO_2_ sample synthesized by the sol–gel method with the Cu dopant, we observe prominent peaks at approximately 1 keV and 8 keV, indicative of the presence of Cu [66,67,68]. In the EDX spectra, a distinct peak at approximately 2.4 keV is observed, signifying the presence of sulfur (S) [69,70,71], which again remained in the sample due to incomplete washing of the reaction products and, in particular, from the acidic residue of copper sulfate. The peaks of aluminum and chlorine are also present in the EDX spectra of all doped samples, as in the case of the pure powders at 1.5 and 2.7–2.8 keV, respectively.

The primary distinction between the sol–gel and hydrothermal TiO_2_ samples lies in the intensity of the EDX spectra. Overall, the intensity of the EDX spectra is higher for the sol–gel TiO_2_ samples compared to the hydrothermal TiO_2_ ones, for both the pure TiO_2_ and REE- and Cu-doped TiO_2_ samples. This difference may be attributed to the sol–gel sample having a smoother agglomerate surface than the hydrothermal samples.

In the case of pure TiO_2_, the peaks for Al and Cl inclusions are much more prominent for the hydrothermal sample than for the sol–gel sample.

For the REE-doped TiO_2_ samples, the situation is reversed compared to the pure samples. Although the intensity of the Cl peak becomes relatively identical for both the sol–gel and hydrothermal samples, the intensity of the Al peak is significantly higher for the sol–gel sample than for the hydrothermal sample. Furthermore, the intensity of the REE dopant peaks (Er, Yb) is more prominent in the hydrothermal sample TiO_2_ than in the sol–gel sample.

Similarly, in the case of Cu-doped TiO_2_, the situation reflects that of the REE-doped samples. The intensity of the Cl peak is relatively similar for both the sol–gel and hydrothermal samples, while the Al peak is more prominent for the sol–gel sample. The S peak is more intense for the hydrothermal sample. Like the REE-doped TiO_2_ samples, where the REE dopant peak intensity was higher for the hydrothermal sample, in the case of the Cu-doped sample, the Cu dopant peaks are also more intense for the hydrothermal sample than for the sol–gel sample.

In summary, the EDX spectra intensity is higher for the sol–gel TiO_2_ samples. The Al and Cl peak intensity in the case of the pure TiO_2_ samples is higher for the hydrothermal sample, while after doping (observed for both REE and Cu dopants), the situation is reversed, as these peaks become more intense for the sol–gel sample. Meanwhile, the dopant (both REE and Cu) peaks are more prominent for the hydrothermal samples. The variation in the intensity of peaks can be reasoned by either the concentration of the elements in the TiO_2_ powders or the EDX measurement errors.

### 3.4. XPS Surface Composition Analysis of TiO_2_ Samples

**Figure 7:**
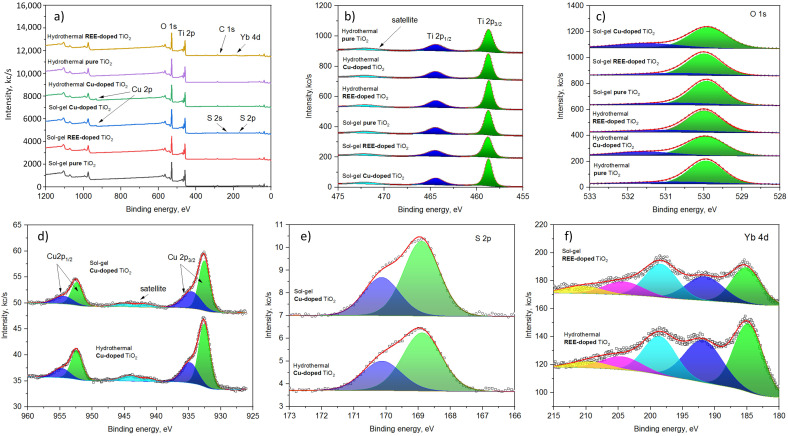
XPS (**a**) survey and (**b**–**f**) narrow spectra of the pure and Cu- and REE-doped TiO_2_ NP samples formed by hydrothermal and sol–gel methods.

The XPS survey spectra are shown in Figure 7a. The main bands corresponding to Ti 2p, O 1s, Cu 2p, S 2p, and Yb 4d have been identified and measured at a higher resolution, as depicted in Figure 7a–e.

The spectra of Ti 2p contain bands at 458.7 eV, 464.5 eV, and 472.0 eV corresponding to 2$p_{3/2}$, 2$p_{1/2}$, and satellite features (Figure 7a). The spectral positions of the bands are identical for all samples studied and are attributed to ${Ti}^{4+}$ [72].

The spectra of O 1s for different samples are similar (Figure 7b); they contain a strong signal at 529.9 eV (Ti-O) and small intensity bands in the range of 531.2–531.6 eV, corresponding to surface oxidation and hydroxyl groups [73].

The signal of copper for sol–gel and hydrothermally synthesized samples is also characterized by almost the same pattern (Figure 7c). They contain two groups of peaks that appear in the range of 933 eV (Cu 2$p_{3/2}$) to 955 eV (Cu 2$p_{1/2}$). Additionally, a smaller intensity signal present in the range of 940–945 eV corresponds to satellite features. The position and relative intensity of the satellite feature suggest that both ${Cu}^{1+}$- and ${Cu}^{2+}$-containing compounds are present in the material [74,75].

The detailed analysis of the Cu 2$p_{3/2}$ region reveals the peaks present at 932.6 eV and 935.0 eV (hydrothermal) and 932.6 eV and 934.6 eV (sol–gel). The smaller energy peaks correspond to to copper oxide, while the higher intensity peaks correspond to ${CuSO}_{4}$ [76]. The latter is supported by the sulfur signal present in the spectra of the copper-containing samples at 168.9 eV (2$p_{3/2}$) and 170.1 eV (${2p}_{1/2}$) that correspond to sulfate groups (Figure 7e) [77].

The detailed analysis of Yb 4d spectra reveals a complex structure of signals, with peaks appearing at 184.7 eV (185.1 eV), 191.7 eV (191.6 eV), 198.5 eV (198.4 eV), 204.5 eV (204.2 eV), and 210.0 eV (210.5 eV) for hydrothermal (sol–gel) samples. The spectral positions of the principal components suggest that the dominant valence state of Yb is 3+ [78].

## 4. Conclusions

Pure TiO_2_ and Cu- or REE-doped TiO_2_ samples were synthesized by sol–gel, hydrothermal, and extraction pyrolysis methods to comprehensively study their structure. The characterization of the samples was accomplished using complementary techniques of Raman spectroscopy, scanning electron microscopy, energy-dispersive X-ray spectroscopy, and X-ray photoelectron spectroscopy.

Raman spectroscopy revealed a gradual anatase–rutile transition for the pure EP samples with an increase in the annealing temperature from 500 to 750 °C, which correlates with previously reported data for sol–gel and hydrothermal samples. Raman spectroscopy is shown to be sensitive enough to the slightest content of the rutile phase in the transition temperature range from 400 to 500 °C in the pure and REE- and Cu-doped titanium oxide powders.

SEM analysis showed that all of the pure and doped titanium oxide samples consist of microsized agglomerates composed of nanoparticles. Sol–gel nanoparticles are characterized by a rather smooth surface, while hydrothermal ones have prominent faces and edges. REE doping leads to some increase in the nanoparticle size. SEM characterization also revealed a more densely packed surface of the hydrothermal samples. The larger nanoparticles are thought to be more favorable for the capture of molecules from the environment and less susceptible to by-products that are washed from them.

The methods of elemental and phase analysis used proved to be suitable techniques for detecting undesirable contaminants in the TiO_2_ samples depending on the synthesis approach. Such contaminants may be present as a result of the structural features of titanium oxide nanoparticles, the insufficient washing of reaction products, and the capture of foreign molecules from the environment. Raman spectroscopy is more sensitive to residues of organic compounds, which is confirmed by pronounced signatures in the region of 700–1200 cm^−1^. However, it does not detect inclusions of chlorine and sulfur as well as intentionally introduced dopants. On the other hand, EDX and XPS sense inclusions invisible to Raman spectroscopy. More importantly, complex analysis by these methods allows us to draw conclusions about the surface or internal arrangement of inclusions in titanium oxide nanoparticles. In more detail, EDX “feels” the chlorine content, in contrast to XPS. Considering the penetration of the electron beam to a depth of at least 1 µm into the sample, and the higher surface sensitivity of XPS, it can be argued that chlorine evaporates from the surface of titanium oxide nanoparticles but remains in their volume. The sulfur by-product, the REE- and Cu-dopants, are distributed in the volume and on the surface of the titanium oxide nanoparticles.

The implemented characterization approach provided a deeper understanding of how different synthesis techniques and dopants affect the properties of TiO_2_ nanoparticles. It should be pointed out that our study was not primarily intended to help with choosing a particular method, but to offer a broader range of options. Bearing in mind the results obtained, one using a particular method is aware of the nuances of both the surface and bulk morphology of titanium oxide nanoparticles and, what is more important in technological terms, understands how the environment and synthesis regimes affect these properties. The problem solved in our work is quite important for researchers involved in the selection of regimes for the formation of pure and doped titanium oxide with specified structural properties. For the first time, we provide a rich characterization of the structure of titanium oxide formed by three different methods, right up to the differentiation of surface and volumetric features of semiconductor nanoparticles.

## Figures and Tables

**Table 4:** Identification of Raman spectra peaks for pure titanium dioxide NPs.

Phase	Peak Position, cm^−1^						Assignment
	Extraction–Pyrolytic (500 °C)	Extraction–Pyrolytic (600 °C)	Extraction–Pyrolytic (650 °C)	Extraction–Pyrolytic (750 °C)	Sol–Gel	Hydrothermal	
Anatase	143	143	143	-	144	143	E_g_
Rutile	-	-	-	145	-	-	B_1g_
Anatase	197	192	197	-	196	197	E_g_
Rutile	-	-	226	239	-	-	Second-order scattering
Anatase	395	395	395	-	395	395	B_1g_
Rutile	-	440	443	442	-	-	E_g_
Anatase	517	515	515	-	518	515	Doublet of B_1g_ and A_1g_
Rutile	-	-	605	607	-	605	A_1g_
Anatase	636	634	636	-	639	636	E_g_
-	-	-	-	-	-	782	Undefined
-	-	-	-	-	-	1073	C–H, C–O, C–C groups
-	-	-	-	-	-	1117	

**Table 5:** Identification of Raman spectra peaks in REE-doped titanium dioxide.

Phase	Peak Position, cm^−1^		Assignment
	Sol–Gel	Hydrothermal	
Anatase	146	144	E_g_
	196	196	
	243	237	
Rutile	323	-	Second-order scattering
	360	-	
Anatase	398	395	B_1g_
	517	518	Doublet of B_1g_ and A_1g_
Rutile	598	597	A_1g_
Anatase	640	633	E_g_
-	702	701	Ti–OH
-	770	769	Undefined
-	1093	1076	C–H, C–O, C–C groups
-	1124	1115	

**Table 6:** Identification of Raman spectra peaks in Cu-doped titanium dioxide.

Phase	Peak Position, cm^−1^		Assignment
	Sol–Gel	Hydrothermal	
Anatase	147	145	E_g_
	195	197	
Anatase	396	395	B_1g_
	515	515	Doublet of B_1g_ and A_1g_
Anatase	640	638	E_g_

## Data Availability

Data are contained within the article. Raw data available on request.

## Abbreviations

The following abbreviations are used in this manuscript:

REE	Rare Earth Elements
XPS	X-ray Photoelectron Spectroscopy
SEM	Scanning Electron Microscopy
EDX	Energy-Dispersive X-ray Spectroscopy
DFT	Density Functional Theory
TTIP	Titanium Tetraisopropoxide
EP	Extraction–Pyrolytic Method
LDA	Local Density Approximation
